# The Left Amygdala and Right Frontoparietal Cortex Support Emotional Adaptation Aftereffects

**DOI:** 10.3390/brainsci14030257

**Published:** 2024-03-06

**Authors:** Xinqi Su, Ruilin Fu, Huiling Li, Nan Jiang, Aqian Li, Jingyu Yang, Leilei Mei

**Affiliations:** 1 Philosophy and Social Science Laboratory of Reading and Development in Children and Adolescents, South China Normal University, Ministry of Education, 510631 Guangzhou, China; 2021023750@m.scnu.edu.cn (X.S.);; 2 School of Psychology, South China Normal University, 510631 Guangzhou, China; 3 Guangdong Key Laboratory of Mental Health and Cognitive Science, South China Normal University, 510631 Guangzhou, China; 4 School of Psychology, Guizhou Normal University, 550025 Guiyang, China

**Keywords:** adaptation aftereffects, emotion, facial expression, amygdala, fMRI

## Abstract

Adaptation aftereffects—in which prolonged prior experience (adaptation) can bias the subsequent judgment of ambiguous stimuli—are a ubiquitous phenomenon. Numerous studies have found behaviorally stable adaptation aftereffects in a variety of areas. However, it is unclear which brain regions are responsible for this function, particularly in the case of high-level emotional adaptation aftereffects. To address this question, the present study used fMRI technology to investigate the neural mechanism of emotional adaptation aftereffects. Consistent with previous studies, we observed typical emotional adaptation effects in behavior. Specifically, for the same morphed facial images, participants perceived increased sadness after adapting to a happy facial image and increased happiness after adapting to a sad facial image. More crucially, by contrasting neural responses to ambiguous morphed facial images (i.e., facial images of intermediate morph levels) following adaptation to happy and sad expressions, we demonstrated a neural mechanism of emotional aftereffects supported by the left amygdala/insula, right angular gyrus, and right inferior frontal gyrus. These results suggest that the aftereffects of emotional adaptation are supported not only by brain regions subserving emotional processing but also by those subserving cognitive control.

## 1. Introduction

Adaptation aftereffect is a phenomenon where the perception of stimuli, especially stimuli with ambiguous features, may be biased towards non-adapted features after adaptation to a recently seen feature [1,2,3]. Research on adaptation aftereffects initially concentrated on simple manipulations such as color, orientation, and motion direction [4,5,6,7]. Recently, a growing number of studies have found that aftereffects also affect high levels of perception, including gender [8,9,10], race [8,11], identity [12,13,14,15,16], and emotion [17,18,19,20,21].

For high-level adaptation, emotional adaptation aftereffects have received great attention because they are critical for species survival as well as individual quality of life [22,23,24,25]. Emotional adaptation aftereffects are typically investigated by using a task in which participants are presented with an emotional stimulus (i.e., adapting stimulus) first and then judging the emotional category of the subsequent morphed stimuli. The phenomenon where participants tend to judge the emotions of morphed stimuli in the opposite direction to those of adapting stimuli is called the emotional adaptation aftereffect. For instance, long-term exposure to a happy face can lead to a neutral face appearing sad afterwards [20]. By using the emotional adaptation paradigm, numerous studies have shown that emotional adaptation aftereffects can be consistently elicited in different populations, regardless of their age, gender, and race [17,21,22,26,27,28,29]. Moreover, emotional adaptation aftereffects are quite robust, even when the duration of adaptation is very short [30,31] or the faces used as adapting stimuli are outside awareness [32,33] or covered [23,28,34]. In addition, emotional adaptation aftereffects occur across identities [22,35,36,37,38,39], levels [40,41], and modalities [20,42,43,44,45,46,47].

Despite the accumulating behavioral evidence for emotional adaptation aftereffects, the understanding of the neural mechanisms is relatively sparse. For example, utilizing high-temporal-resolution electroencephalography (EEG) technologies, Cheal et al. [17] found that after subjects adapted to an emotional stimulus (e.g., a happy or sad facial image), physically identical neutral test stimuli not only showed a behavioral perceptual bias (i.e., emotional adaptation aftereffect) but also resulted in N170 latency differences. The emotional adaptation effect has also been reported in the form of an increased late component between 300 and 400 ms in a magnetoencephalography (MEG) study [48]. In addition, Wang et al. [20] investigated cross-modal emotional adaptation aftereffects and found robust P1-N170-N2-P_late_ event-related potential (ERP) waveforms in both hemispheres. These results indicate that emotional adaptation aftereffects occur in both the early and late stages of emotion processing. Nevertheless, due to the relatively low spatial resolution of EEG and MEG technologies, it is still unclear which brain regions are responsible for emotional adaptation aftereffects.

In contrast, functional magnetic resonance imaging (fMRI) technology offers superior spatial resolution, enabling brain activity to be pinpointed with greater anatomical precision, making it possible to examine which brain regions are associated with emotional adaptation aftereffects. To our knowledge, only one study has explored the neural mechanisms of emotional adaptation effects by using fMRI. Specifically, Furl and colleagues [1] examined how brain activity associated with the perception of expression and identity categories in ambiguous morphed faces was influenced by prior adaptation. For expression categorization, the adaptation aftereffect was associated with heightened right medial temporal cortex activity, specifically when subjects perceived the non-adapted emotion category [1]. Notably, happy and fearful adaptation conditions, which might elicit opposite aftereffects [18], were combined as a single condition to contrast with the non-adapted condition in this study. This design would probably cancel out the two opposite effects. Therefore, brain regions related to emotional adaptation aftereffects should be further specified.

To explore brain regions for emotional adaptation aftereffects, we used fMRI technology and the classic emotional adaptation paradigm in which we first presented participants with an emotional facial image and then had them judge the facial expression of the subsequent morphed image. For behavioral data, we used a psychometric function to fit the subjects’ behavioral responses. We hypothesized that participants would tend to judge the subsequent morphed images as sad after adapting to a happy facial image or judge them as happy after adapting to a sad facial image. With the imaging data, we examined the neural mechanisms of emotional adaptation aftereffects by performing whole-brain activation analysis, in which the neural responses to test images with ambiguous expressions were compared across the happy, neutral, and sad adaptation conditions (i.e., stimulus-based analysis). These results were further confirmed by conducting a perception-based region-of-interest (ROI) analysis (i.e., the happy, neutral, and sad conditions were defined based on subjects’ responses). Based on previous ERP findings of emotional adaptation affecting both early and late components [17,20,48], we expected that emotional adaptation effects would be supported by brain regions subserving high-level cognitive control (e.g., frontoparietal regions) in addition to those subserving emotional processing (e.g., the amygdala).

## 2. Materials and Methods

### 2.1. Participants

Twenty-two native Chinese participants (mean age = 20.73 ± 2.25 years; six males) were recruited for this study. To ensure the suitable sample size for our experiment to detect effects, we used G*Power 3.1 [49] to conduct power calculations and found that 17 participants are sufficient for medium effect size (i.e., 0.25) with 0.80 power in one-way repeated-measures analysis of variance [50,51]. All participants were right-handed (mean = 73.45, SD = 18.49) [52], had normal or corrected-to-normal vision, and self-reported no history of neurological or psychiatric disorders. Before the experiment, written informed consent was obtained from all participants. All experimental procedures were approved by the Institutional Review Board of the School of Psychology at South China Normal University (SCNU-PSY-319, approved on 19 November 2018) and conducted in accordance with the relevant regulations of the Institutional Review Board. One participant was excluded from the subsequent analysis because of an excessively high rate of nonresponse trials (25.93%, fell outside of three standard deviations from the mean) during scanning.

### 2.2. Materials

**Figure 1:**
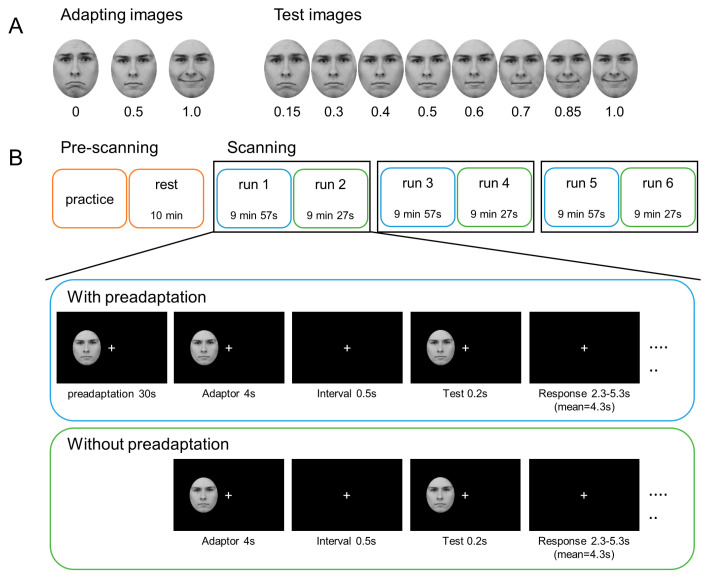
Stimuli and experimental design. (**A**) Three adapting images (i.e., sad, neutral, and happy adaptors) and eight morphed test images (i.e., happy proportion equals 0.15, 0.3, 0.4, 0.5, 0.6, 0.7, 0.85, 1.0). The original images of three adaptors were obtained from the KDEF database [53] (http://www.emotionlab.se/resources/kdef, accessed on 5 September 2018). All test images utilized in the experiment were created by WebMorph (STOIKimage, https://webmorph.org, accessed on 5 September 2018) according to the three adaptors. (**B**) General experimental procedure outline and single trial procedure. The orange box represented the program before scanning. Blue and green boxes represented the first and second runs with and without 30 s of preadaptation under each adaptor condition, respectively.

Three facial images showing happy (happy proportion equals 1), neutral (happy proportion equals 0.5), and sad (happy proportion equals 0) expressions on one male person were selected from the Karolinska Directed Emotional Faces database (KDEF) [53] as adapting images. All test images used in the experiment were generated using WebMorph (STOIKimage, https://webmorph.org, accessed on 5 September 2018). In line with prior research [20,21,22], we employed a morphing technique to blend the sad facial image with the neutral facial image, resulting in a series of images exhibiting varying proportion of happiness ranging from 0 to 0.5. Similarly, we morphed the neutral image with the happy image to create another series of images from 0.5 to 1. In the current experiment, eight images with proportions of happiness equal to 0.15, 0.3, 0.4, 0.5, 0.6, 0.7, 0.85, and 1.0 were chosen as test images to generate robust and informative psychometric curves (Figure 1A). All adapting and test images were converted to grayscale and cropped into an oval shape to remove external features. In addition to the eight test images, two grating images titled 45° to the left or right of the vertical axis were included for other purposes.

We recruited another 16 Chinese participants to evaluate the emotion category (sad, neutral, or happy) and degree of arousal (1 = “very low”, 7 = “very high”) associated with each image. The results showed that the three adapting images fit into human emotional perception of happiness, neutrality, and sadness and that the degrees of arousal associated with the eight test images coincided with people’s subjective judgments (see Figure S1). Specifically, the rating scores of emotional facial images were higher than those of neutral images, and the degrees of arousal associated with all images followed a U-shaped curve.

### 2.3. Procedure

#### 2.3.1. Pre-Scanning

Before the formal fMRI scan, participants underwent a brief practice session to familiarize themselves with the experimental procedure. The images utilized in the practice session were not presented during the fMRI scan. After the practice, all participants were instructed to rest for 10 min to keep them in a calm state for a subsequent fMRI scan. All behavioral data were compiled using Psychtoolbox-3 (http://www.psychtoolbox.org/, accessed on 8 June 2015) in MATLAB R2013a (https://www.mathworks.com, accessed on 8 July 2018).

#### 2.3.2. Scanning

The fMRI scan consisted of three adaptor conditions (i.e., neutral, sad, and happy adaptor conditions). Each condition consisted of two functional runs. For the first run of each condition, an extra 30 s preadaptation was included to enhance the adaptation effect and prevent interference from the preceding adaptor condition. The order of the three conditions was counterbalanced across participants. Each run consisted of 63 trials, with 7 repetitions for each of the 8 test images and the grating image. The trials were presented in a pseudorandom manner, and OPTSEQ2 (http://surfer.nmr.mgh.harvard.edu/optseq/, accessed on 8 June 2021) was applied to optimize the trial sequences.

#### 2.3.3. Trial Procedure

Each trial began with the presentation of an adapting image for 4 s, succeeded by a 0.5 s fixation interval (interstimulus interval, ISI). Next, a test image was presented for 0.2 s (Figure 1B). Participants were instructed to accurately and swiftly determine whether the test image was happy or sad or whether the orientation of the grating image leaned towards the left or right by pressing one of two keys (the “1” key for happy or left and the “4” key for sad or right). The key assignment was counterbalanced among participants throughout the experiment. Participants were instructed to fixate on a white cross in the center of the black screen to eliminate the effects of fixation differences [30,54]. For each trial, the response time was a random jitter (i.e., fixation) lasting between 2.3 and 5.3 s (mean = 4.3 s) to enhance the design efficiency [55]. Additionally, on the basis of previous findings suggesting stronger adaptation aftereffects when the stimuli are presented in the visual periphery than at the fovea [20,28], all images were presented on the left side of the central fixation cross throughout the experiment.

### 2.4. Acquisition of Imaging Data

The MRI data were acquired using a 3.0 T Siemens MRI scanner located at the MRI Center at South China Normal University. Functional images were obtained through a single-shot T2*-weighted gradient-echo echo-planar imaging (EPI) sequence (58 axial slices, repetition time (TR)/echo time (TE)/θ = 2000 ms/30 ms/90°, field of view (FOV) = 224 × 224 mm, matrix size = 112×112, slice thickness = 2.0 mm), resulting in a voxel size of 2.0 × 2.0 × 2.0 mm. Anatomical images were acquired with a T1-weighted three-dimensional gradient-echo pulse sequence (176 sagittal slices, TR/TE/θ = 2530 ms/1.94 ms/7°, FOV = 256 × 256 mm, matrix size = 256 × 256, slice thickness = 1.0 mm). Anatomical magnetization-prepared rapid gradient-echo (MPRAGE) images were collected with 0.5 × 0.5 × 1.0 mm resolution.

### 2.5. Analysis of Behavioral Data

To measure the emotional adaptation aftereffects, we initially computed the fraction of happy responses to the individual test images within each adaptation condition for every participant. Subsequently, the fractions from all participants were averaged to obtain the mean happy response fractions of each test image across the three adaptation conditions. Next, the mean fractions of happy responses were plotted against the proportions of happiness in the morphed test image. These results were then fitted with a sigmoidal function for each condition in the form of *f*(*x*) = 1/[1 + e^−*a*(*x*−*b*)^], where *b* represents chance performance [50% point of the psychometric function, i.e., the point of subjective equality (PSE)] and *a*/4 determines the slope of the function at the PSE. The amplitude of the adaptation aftereffect was equal to the PSE of emotional adaptation (happy or sad adaptor) condition minus the baseline (neutral adaptor) condition. The positive and negative values represent the psychometric curve shifting to the right and left, respectively. In other words, more or less happiness judgments were made compared to the baseline. Finally, the significance of the adaptation aftereffect was assessed through one-sample t tests.

### 2.6. Image Preprocessing and Activation Analysis

Image preprocessing was performed using FEAT (FMRI Expert Analysis Tool) Version 6.00 in FSL (FMRIB’s Software Library, http://www.fmrib.ox.ac.uk/fsl, accessed on 3 March 2020). To achieve T1 signal equilibrium, the first four volumes in each time series were discarded. Subsequently, the remaining images were stripped of non-brain tissue using the brain extraction tool [56] and realigned using MCFLIRT [57]. All participants were confirmed to have no translational movement parameters exceeding 1 voxel in any direction for any run. A 5 mm full-width-at-half-maximum (FWHM) Gaussian kernel and a nonlinear high-pass filter with a cutoff of 100 s were, respectively, used for spatial smoothing and temporal filtering of the functional data. A two-step registration process was employed to register the functional images to standard Montreal Neurological Institute (MNI) space: first from the functional images to the MPRAGE structural images and then to the MNI template [58]. The second step of registration was further refined using FNIRT non-linear registration [59,60].

The analysis was conducted in three levels. At the first level, a general linear model (GLM) was applied to the preprocessed data for each participant and each run. The regressors for the GLM were generated by convolving the event onsets and durations with a double-gamma hemodynamic response function. According to previous research [1,20], we divided the 8 test images into three conditions: happy (proportion of happiness: 0.7, 0.85, and 1.0), neutral (proportion of happiness: 0.4, 0.5, and 0.6), and sad (proportion of happiness: 0.15 and 0.3). The preadaptation images, adapting images, and grating images were modeled as nuisance variables to avoid their potential confounding effects. The fixation was not explicitly modeled and consequently served as an implicit baseline. To improve statistical sensitivity, the 6 fundamental motion parameters and their temporal derivatives were included as covariates of no interest. Following previous studies [1,17], we focused on the effects of emotional adaptation on the perception of images with ambiguous expressions (i.e., the neutral condition) in the subsequent analysis. Thus, the contrast of the neutral condition was computed for each run and each participant.

A second-level analysis was then conducted for each participant by concatenating the imaging data from all six runs using a fixed-effects model. In the third-level analysis, group activations were obtained by using Simple OLS (ordinary least square, FSL’s local analysis of mixed effects). All reported group images were thresholded with a height threshold of Z > 2.6 (i.e., *p* < 0.005) and a cluster probability of *p* < 0.05, corrected for whole-brain multiple comparisons using Gaussian random field theory [61].

### 2.7. ROI Analysis

An ROI analysis was further performed to confirm the results of the above stimulus-based analysis [i.e., the same set of morphed facial images (proportion of happiness: 0.4, 0.5, and 0.6) was used in the three adaptation conditions]. In the ROI analysis, a perception-based analysis was used because there is evidence that neural activation during the adaptation paradigm depends on the perceptual bias of participants [17,62,63] and that adaptation aftereffects are more pronounced in perception-based analysis than in stimulus-based analysis [13]. Following previous studies [13,17], three perception-based conditions were constructed: neutral test trials following a neutral adapting image (hereinafter, neutral tests following a neutral adaptor), neutral trials with sad perception following a happy adapting image (hereinafter, sad perception following a happy adaptor) and neutral trials with happy perception following a sad adapting image (hereinafter, happy perception following a sad adaptor).

A total of 4 ROIs were defined in the current study. First, to further confirm the results of the stimulus-based analysis, three ROIs (i.e., the left amygdala/insula, right angular gyrus, and right inferior frontal gyrus) were functionally defined based on the activation clusters found in whole-brain activation analysis. In addition, to explore the effect of face perception on emotional adaptation aftereffects, the right fusiform face area (FFA), a key brain region for face perception, was additionally defined as a sphere of 6 mm radius around the coordinates (MNI: 40, −55, −10) reported in Kanwisher et al. [64].

In the abovementioned four ROIs, percent signal changes were then calculated by using the following formula: [contrast image/(mean of run)] × ppheight × 100%. Specifically, the contrast image was extracted from each perception-based condition of the fitted GLM. The mean of the run was calculated as the mean of the functional data of the fitted GLM. The variable “ppheight” represented the peak height of the hemodynamic response versus the baseline level of activity [65]. Finally, one-way repeated-measures analysis of variance (ANOVA) was performed on all ROIs separately to investigate the differences among conditions. If the main effect was significant, Bonferroni post hoc test was performed.

## 3. Results

### 3.1. Behavioral Results

**Figure 2:**
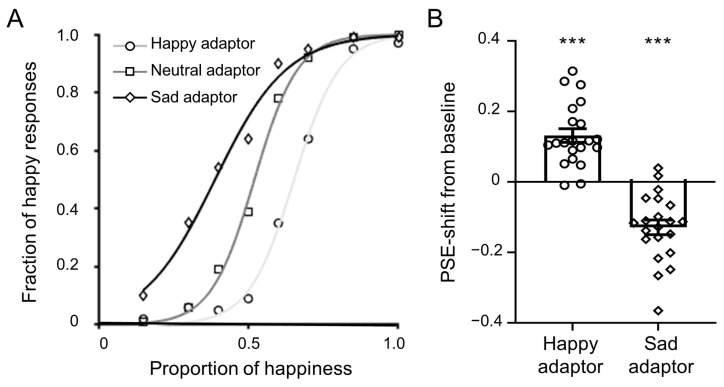
The emotional adaptation aftereffect in behavior. (**A**) The fraction of happy responses is plotted as a function of the proportion of happiness of the test images, separately for the happy (*R*^2^: 0.98 ± 0.03), neutral (*R*^2^: 0.99 ± 0.01), and sad (*R*^2^: 0.97 ± 0.04) adaptor conditions; (**B**) the mean PSE shift of the happy and sad adaptor conditions from the neutral adaptor condition for all participants. Error bars represent standard errors. *** *p* < 0.001.

The behavioral responses in the three adaptation conditions are illustrated in Figure 2A. The sigmoidal function fitted the participant responses well, with *R*^2^ values (mean ± SD) of 0.98 ± 0.03 in the happy, 0.99 ± 0.01 in the neutral, and 0.97 ± 0.04 in the sad adaptor conditions. After adapting to the happy facial image, participants perceived happy expressions less frequently, and the psychometric curve shifted to the right compared to the neutral adaptor. After adapting to the sad facial image, participants perceived happy expressions more frequently, and the psychometric curve shifted to the left compared to the neutral adaptor.

To quantify the aftereffects, we calculated the PSE shift relative to the baseline condition (neutral adaptor) for the happy and sad psychometric curves of all participants. As demonstrated in Figure 2B, both the happy adaptor (mean PSE shift = 0.13, *t* (20) = 6.77, *p* < 0.001, Cohen’s *d* = 3.03) and the sad adaptor (mean PSE shift = −0.13, *t* (20) = −6.15, *p* < 0.001, Cohen’s *d* = −2.75) produced significant adaptation aftereffects. Specifically, compared to the baseline condition, for test stimuli with identical physical properties, participants made opposite reactions under happy and sad adaptation conditions, i.e., more frequent responses to sadness and happiness, respectively.

### 3.2. fMRI Results

**Figure 3:**
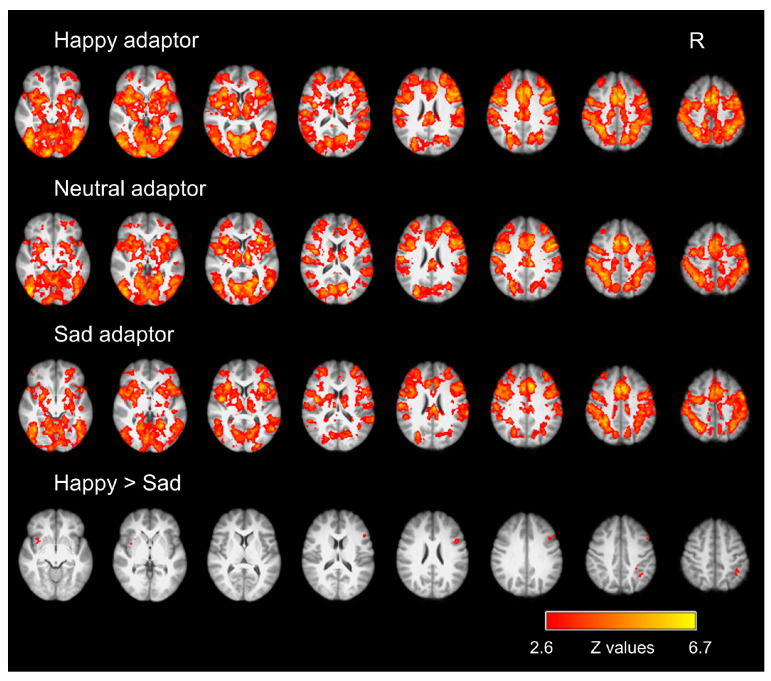
Brain regions showing different neural activations across the three types of adaptor conditions (i.e., happy, neutral, and sad) during emotion judgment on the neutral test images. All activations were whole-brain-corrected and -thresholded at Z > 2.6. R = right.

Whole-brain activation analysis was conducted to explore neural activations for emotional adaptation aftereffects. Following previous studies, we focused on the images with intermediate morph levels (i.e., the neutral images) in this analysis because the emotional adaptation aftereffect was greatest for test stimuli with intermediate morph levels (see Figure 2). Activation analysis revealed that all three adaptation conditions elicited activation in an extensive neural network compared to the fixation, including the bilateral insular cortex, superior frontal gyrus, middle frontal gyrus, inferior frontal gyrus, precentral gyrus, postcentral gyrus, superior parietal lobule, supramarginal gyrus, lateral occipital cortex, supplementary motor cortex, cingulate gyrus, precuneus, lingual gyrus, and fusiform cortex (Figure 3). Further comparisons between different adaptation conditions revealed that the happy adaptation condition showed stronger activation than the sad adaptation condition in the left amygdala/insula, right angular gyrus, and right pars opercularis of the inferior frontal gyrus (Figure 3). No significant activation differences were found for other contrasts.

**Figure 4:**
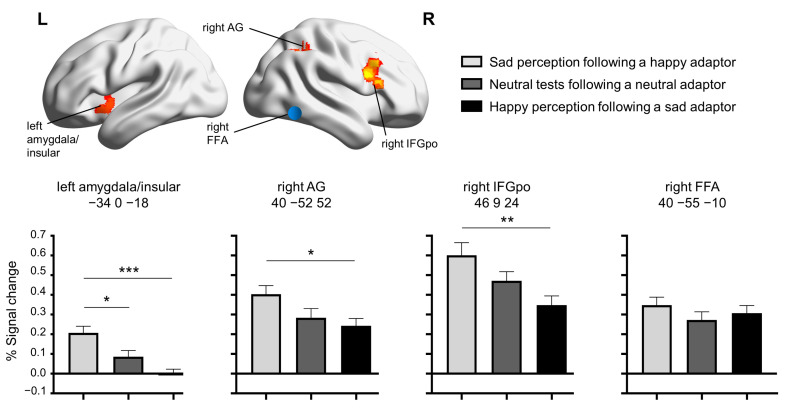
The percent signal changes of the three perception-based conditions in 4 ROIs. * *p* < 0.05, ** *p* < 0.01, and *** *p* < 0.001. Abbreviations: AG, angular gyrus; IFGpo, pars opercularis of inferior frontal gyrus; FFA, fusiform face area.

Perception-based ROI analysis was also conducted to confirm the activation results found in the stimulus-based analysis described above. As mentioned in the Methods, the three perception-based conditions (i.e., sad perception following a happy adaptor, neutral tests following a neutral adaptor, and happy perception following a sad adaptor) were constructed based on participants’ subjective perception. The results showed significant emotional adaptation aftereffects in the three brain regions identified in the aforementioned whole-brain analysis, but no such effects were found in the brain region responsible for face processing (i.e., the right FFA; *F* (2, 60) = 0.87, *p* = 0.424, *η*^2^*_p_* = 0.028) (Figure 4). Specifically, the main effect of emotional adaptation was significant in the left amygdala/insula (*F* (2, 60) = 12.63, *p* < 0.001, *η*^2^*_p_* = 0.296; q_FDR_ < 0.001), right angular gyrus (*F* (2, 60) = 3.72, *p =* 0.03, *η*^2^*_p_* = 0.110; q_FDR_ = 0.04), and right pars opercularis of the inferior frontal gyrus (*F* (2, 60) = 5.65, *p* = 0.006, *η*^2^*_p_* = 0.158; q_FDR_ = 0.012). Post hoc comparisons revealed significantly higher activation in the perception-based happy condition than in the perception-based sad condition in all three ROIs (left amygdala/insula: *t* (20) = 5.18, *p* < 0.001, Cohen’s *d* = 1.64; right AG: *t* (20) = 2.76, *p* = 0.033, Cohen’s *d* = 0.87; right IFGpo: *t* (20) = 3.19, *p* = 0.004, Cohen’s *d* = 1.01) and in the perception-based neutral condition in the amygdala/insula (*t* (20) = 2.65, *p* = 0.014, Cohen’s *d* = 0.84; Figure 4).

## 4. Discussion

Using fMRI technology and the classic emotional adaptation paradigm, this study examined the neural mechanism of emotional adaptation aftereffects. Consistent with previous studies [18,21,22,23,28,66,67], the behavioral results showed a typical emotional adaptation aftereffect. Specifically, participants tended toward sad judgments of the subsequent morphed images after adapting to a happy facial image and tended toward happy judgments after adapting to a sad facial image. More importantly, imaging results showed that emotional adaptation aftereffects were found in brain regions subserving emotion processing (left amygdala/insula) and cognitive control (right inferior frontal gyrus pars opercularis and right angular gyrus), but not in brain regions subserving face perception (e.g., the right fusiform face area). These findings imply that a priori adaptation, which behaviorally biases emotional judgments against non-adapted categories, requires high-level emotion processing and cognitive control processing.

Our study provides precise spatial locations of brain regions associated with emotional adaptation aftereffects. As discussed in the Introduction, much behavioral research has shown the existence of emotional adaptation aftereffects [18,21,22,23,28,66,67], and numerous electrophysiological studies have examined the time course of these aftereffects [17,20,48]. Here, we specified which brain regions support emotional adaptation aftereffects. Specifically, by comparing neural responses to ambiguous morphed facial images (i.e., facial images of intermediate morph levels) following different adapting emotions, the present study revealed that the same facial images produced greater activation in the left amygdala/insula, right angular gyrus, and right inferior frontal gyrus after adapting to a happy facial image than after adapting to a sad facial image. These results were further confirmed by taking participants’ behavioral responses into account (i.e., perception-based ROI analysis). In contrast, emotional adaptation aftereffects were not found in the key brain region for face perception (i.e., the right fusiform face area). These results indicate that the emotional adaptation aftereffects are supported by brain regions subserving emotional processing and cognitive control but not by those subserving face perception.

The amygdala, especially the left amygdala, has long been thought to play an important role in emotional processing [68,69,70]. Furthermore, a meta-analysis found that the left amygdala was the brain structure consistently recruited by emotional decision-making, regardless of task instructions [71]. As a cortical center for visceral information processing and interoception, the insula is thought to be crucial in both emotional experience and subjective perception [72,73,74]. Furthermore, the insula, as part of the salience network, is important for the rapid detection of personally relevant or otherwise significant emotional cues in the environment [75,76,77]. Our findings showing the involvement of brain regions subserving emotional processing are consistent with previous findings that emotional adaptation aftereffects were associated with high-level emotional processing [22,30,31,54,78] and that the magnitude of emotional adaptation aftereffects was positively correlated with the emotional intensity of the adapting faces [19,27,30,79].

In addition, the inferior frontal gyrus is also considered to have a prominent role in the processing of facial expressions [80,81]. The gray matter volume of the right inferior frontal gyrus was closely related to accurate recognition of facial expressions [82], while a lesion of the structure impairs this function [83]. Building a meta-analytic connectivity model, a meta-analysis comprising 96 fMRI and positron emission tomography (PET) studies recently identified a functionally co-activating neural network that includes brain areas like the amygdala and inferior frontal gyrus [84]. Moreover, the right angular gyrus is part of the lateral parietal cortex and serves as a multimodal integration region [85,86,87,88]. It participates in different cognitive tasks through potential connections to different core cognitive networks [89]. More importantly, the angular gyrus and the inferior frontal gyrus together form part of the frontoparietal control network [90], which subserves cognitive control and is critical for coordinating behavior in a rapid, accurate, and flexible goal-driven manner [91]. The frontoparietal control network flexibly couples with and regulates other functional brain networks according to the goal of the current task [92,93]. Our results showing the involvement of brain regions in the frontoparietal control network suggest the critical role of cognitive control in emotional adaptation aftereffects.

Our results have important implications for the theoretical explanation of emotional adaptation aftereffects. Specifically, our results argue against the view that the angle or orientation of the mouth is sufficient to explain the perceived emotional changes in faces [40,41,94]. Based on this view, we inferred that differential activation across adaptation conditions may be found in low-level face perception brain regions (such as the right FFA). However, our actual findings from the stimulus-based whole-brain activation analysis as well as the perception-based ROI analysis did not support this expectation. Instead, our findings could be explained by the model of cognitive control of emotion. This model held that regulating emotional responses is essentially a process of cognitive control of emotion, and different emotion regulation strategies (i.e., cognitive control processes) will affect some or all stages of emotion production [95]. In line with this model, both the stimulus-based whole-brain activation analysis as well as the perception-based ROI analysis in our study showed the involvement of emotional processing brain regions (the left amygdala/insula) and components of the frontoparietal control network (the right angular gyrus, and right inferior frontal gyrus) in emotional adaptation aftereffects.

Two limitations of this study should be discussed. First, because there was a higher proportion of female participants, the study cannot completely eliminate the impact of gender differences on the neural mechanisms of emotional adaptation. Therefore, future studies should replicate our findings by recruiting participants with a more balanced male-to-female ratio. Second, although behavioral results showed significant differences in PSE between the emotion adaptation conditions (i.e., happy and sad) and the neutral adaptation condition, there were no differences observed in neural activation in any brain region. This discrepancy may be attributable to the relatively small number of trials (i.e., facial images of intermediate morph levels), which might not provide a sufficient signal-to-noise ratio in BOLD responses. Future studies should attempt to replicate our results using a larger number of trials [1,96].

In summary, using a classical emotional adaptation paradigm and fMRI technology, we found that prior adaptation experiences biased emotion judgment toward the non-adapted category. More importantly, emotional adaptation aftereffects were supported by brain regions subserving emotional processing and cognitive control but not by those subserving low-level face perception. These results suggest that emotional adaptation aftereffects are a high-level phenomenon.

## Data Availability

The data that support the findings of this study are available from the corresponding author upon reasonable request. The data are not publicly available due to privacy and ethical restrictions.

## Supplementary Materials

The following supporting information can be downloaded at: https://www.mdpi.com/article/10.3390/brainsci14030257/s1, Figure S1: Emotion classification and arousal evaluation.
