# Supplementary material for: The Left Amygdala and Right Frontoparietal Cortex Support Emotional Adaptation Aftereffects
Source: Brain Sci. 2024 Mar 6;14(3):257. doi: 10.3390/brainsci14030257 (PMC10968625; doi:10.3390/brainsci14030257)
Supplement: Supplementary file 1 [file brainsci-14-00257-s001.zip › brainsci-2877658-supplementary.pdf]

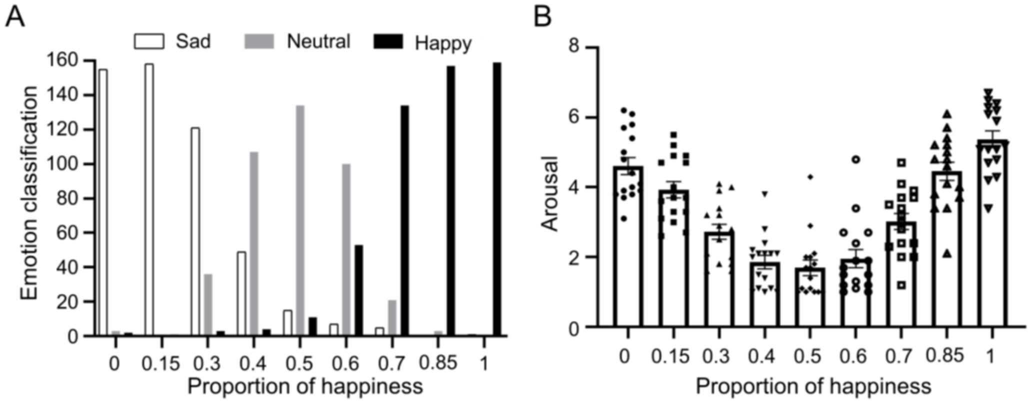

Figure S1. Emotion classification and arousal evaluation. For each facial image, the arousal and emotion categorizations were evaluated 10 times by 16 subjects. (**A**) The frequencies of participants' emotion classification on each facial image. (**B**) The mean scores of participants' arousal evaluation on each facial image (1 = "very low", 7 = "very high"). Error bars represent standard errors.
